# Performance Evaluation of Four-Parameter Models of the Soil-Water Characteristic Curve

**DOI:** 10.1155/2014/569851

**Published:** 2014-05-21

**Authors:** Siti Jahara Matlan, Muhammad Mukhlisin, Mohd Raihan Taha

**Affiliations:** ^1^Department of Civil and Structural Engineering, Faculty of Engineering and Built Environment, Universiti Kebangsaan Malaysia, 43600 Bangi, Selangor, Malaysia; ^2^Civil Engineering Program, School of Engineering and Information Technology, Universiti Malaysia Sabah, 88400 Kota Kinabalu, Sabah, Malaysia; ^3^Department of Civil Engineering, Polytechnic Negeri Semarang, Jl. Professor Soedarto, SH, Tembalang, Semarang 50275, Indonesia

## Abstract

Soil-water characteristic curves (SWCCs) are important in terms of groundwater recharge, agriculture, and soil chemistry. These relationships are also of considerable value in geotechnical and geoenvironmental engineering. Their measurement, however, is difficult, expensive, and time-consuming. Many empirical models have been developed to describe the SWCC. Statistical assessment of soil-water characteristic curve models found that exponential-based model equations were the most difficult to fit and generally provided the poorest fit to the soil-water characteristic data. In this paper, an exponential-based model is devised to describe the SWCC. The modified equation is similar to those previously reported by Gardner (1956) but includes exponential variable. Verification was performed with 24 independent data sets for a wide range of soil textures. Prediction results were compared with the most widely used models to assess the model's performance. It was proven that the exponential-based equation of the modified model provided greater flexibility and a better fit to data on various types of soil.

## 1. Introduction

The soil-water characteristic curve (SWCC) can be defined as the relationship between water content and suction in unsaturated soils. It can be viewed as a continuous sigmoid function describing the water storage capacity of a soil as it is subjected to various soil suctions. The soil-water characteristic curve contains important information regarding the amount of water contained in the pores in any soil suction situation and the pore size distribution related to the stress state in the soil-water [1].

Soil-water characteristic curves are important for groundwater recharge, agriculture, and soil chemistry. They are used to predict the soil-water storage, water supply to plants (field capacity), and soil aggregate stability [2]. These relationships are also of considerable value in geotechnical and geoenvironmental engineering. Unsaturated soil mechanics has primarily utilized SWCC for the estimation of unsaturated soil property functions which are subsequently used in numerical modeling solutions to geotechnical and geoenvironmental engineering problems [3]. SWCC field or laboratory measurements are difficult, expensive, and time-consuming; however, because of its importance and usefulness, many empirical models were developed to describe the SWCC.

A number of mathematical equations have been proposed in the literature to represent SWCCs. Fredlund and Xing [4] and Leong and Rahardjo [5] reviewed a range of proposed soil-water characteristic curve models along with parametric studies. Most of the equations described earlier are empirical in nature and based on the shape of the soil-water characteristic curve. Sillers et al. [6] also summarized numerous mathematical models of the SWCC. The mathematical models presented in their paper can be categorized in a number of ways to illustrate the characteristic equations such as parametric studies, as well as their advantages and disadvantages. Models such as those of Gardner [7], Brooks and Corey [8], Brutsaert [9], Tani [10], McKee and Bumb [11, 12], Van Genuchten [13], Burdine [14], Mualem [15], Kosugi [16], and Fredlund and Xing [4] were analyzed.

The most commonly employed classical retention models are the sigmoid function models by Gardner [7] and Van Genucthen [13] and the power function model by Brooks and Corey [8]. Recently, the lognormal distribution model by Kosugi [17] has gained popularity due to its great flexibility in terms of representing the water content in wet and dry ranges for all soil types. In contrast, the exponential-based models by McKee and Bumb [11, 12] are less commonly used. According to Sillers et al. [6] the problem with these models is that they are relatively less flexible. After conducting statistical assessment of a number of soil-water characteristic curve models, Sillers and Fredlund [18] concluded that the exponential-based models are the most difficult to fit. One reason for the difficulty in performing the fit is the overlap in the domain of each parameter. This is because the parameters of the exponential-based models affect both the shape and the position of the curve. The interdependence of these parameters causes uniqueness difficulties for the fitting routine which increase the number of iterations and the number of trials before convergence to the best-fit parameters. Tani [10], Russo [19], and Ross and Smettem [20] also proposed soil-water retention models. Their model equations are reproduced from Gardner's [21] exponential function for unsaturated hydraulic conductivity when it is incorporated in Mualem's [15] conductivity model. The equations provide reasonable plots for water retention curves; however, the models are less accurate than the other two- or three-parameter models as described by Kosugi et al. [22].

With this in view, therefore, an exponential-based model is proposed to describe the SWCC. The modified equation is similar to those previously reported by Gardner [7] but includes exponential variable. The Gardner model was chosen as a starting-point because of its simplicity and easiness to fit. Another advantage is that the model has parameters that have an independent effect on the soil-water characteristic curve. The performance of the modified Gardner model is then demonstrated on a variety of soil types and the fitting performance in terms of accuracy (RMSE) and linearity (*R* ^2^) is compared with the existing models suggested by Brooks and Corey [8], Van Genuchten [13], and Kosugi [17]. These three existing models were selected in this study because they are widely adopted and cited and also because of their relative simplicity. They also contain four parameters in their model equations.

## 2. Soil-Water Characteristic Curve (SWCC) Models

Models by Gardner [7], Brooks and Corey [8], and Van Genuchten [13], and the lognormal distribution model by Kosugi [17] are some of the notable models found in the literature. All these models are parametric models based upon a pore size distribution function and the capillary theory. They also contain four parameters in their model equations. The equations representing each model along with definitions of the variables used are given as follows.

In general, the normalized water content or a dimensionless water content term, Θ, which is also called effective saturation, *S*_*e*_, will be used to represent the equations associated with the soil-water characteristic curve models. Consider the following:

(1) $$\begin{array}{c} \Theta =\frac{\left(\theta -\theta_{r}\right)}{\left(\theta_{s}-\theta_{r}\right)}, \end{array}$$

where  *θ* is the volumetric water content and *θ*_*s*_ and *θ*_*r*_ are the saturated and residual volumetric water contents, respectively.

### 2.1. Gardner's Model

The Gardner [7] equation was one of the first equations used to model the soil-water characteristic curve. It is a continuous function originally intended as a means of modeling the unsaturated coefficient of the permeability of soil. The equation has now been adapted, however, to model the soil-water characteristic curve. The equation uses two fitting parameters, namely, *a* and *n*. Parameter *a* is related to the inverse of air entry value and the *n* parameter is related to the pore size distribution [6]. Consider the following:

(2) $$\begin{array}{c} \Theta =\frac{1}{1+a\psi^{n}}, \end{array}$$

where *ψ* is the soil suction with a unit of kilopascals (kPa).

By substituting (1) into (2), we obtain the volumetric water content form of the Gardner model as follows:

(3) $$\begin{array}{c} \theta =\theta_{r}+\frac{\theta_{s}-\theta_{r}}{1+a\psi^{n}}. \end{array}$$

The Gardner model has a particularly simple form with few parameters; it is thus convenient to use and has a wide range of applications. However, it cannot accurately describe the soil-water characteristic curve for saturated and near-saturated soils [23].

### 2.2. Brooks and Corey's Model

Brooks and Corey's [8] model is among the earliest equations proposed for the soil-water characteristic curve and remains a popular model where it is in the form of a power-law relationship. The model is given by the following equation:

(4) $$\begin{array}{c} \Theta ={\left[\frac{\psi }{h_{b}}\right]}^{-\lambda }\;\psi >h_{b},\\ \Theta =1\;\psi \leq h_{b}. \end{array}$$

The equation uses two fitting parameters, namely, *h*_*b*_ and *λ*. Parameter *h*_*b*_ is related to the air entry value of the soil. The *λ* parameter is termed the pore size index and is related to the pore size distribution of the soil. The model is assumed to be constant for suctions less than the air entry value. The soil-water characteristic curve is assumed to be an exponential decreasing function at soil suctions greater than the air entry value [6].

By substituting (1) into (4), the volumetric water content form of the Brooks and Corey model can be written as follows:

(5) $$\begin{array}{c} \theta =\theta_{r}+\left(\theta_{s}-\theta_{r}\right){\left[\frac{\psi }{h_{b}}\right]}^{-\lambda }. \end{array}$$

The Brooks and Corey model is relatively simple and thus widely used [23]; but the model does not provide a continuous mathematical function for the entire soil-water characteristic curve [6].

### 2.3. Van Genuchten Model

The most widely adopted alternative to the Brooks and Corey model is that proposed by Van Genuchten [13]. The model uses three fitting parameters, namely, *α*, *n*, and *m*. The Van Genuchten model can mathematically be described as follows:

(6) $$\begin{array}{c} \Theta =\frac{1}{{\left[1+{\left(\alpha \psi \right)}^{n}\right]}^{m}}. \end{array}$$

To simplify and derive closed form equation for unsaturated conductivity based on Mualem [15], the *n* and *m* parameters in the SWCC equation can have a fixed relationship with *m* = (1 − 1/*n*). This suggestion therefore reduces the three-parameter equation of Van Genuchten to a two-parameter SWCC equation:

(7) $$\begin{array}{c} \Theta =\frac{1}{{\left[1+{\left(\alpha \psi \right)}^{n}\right]}^{\left(1-1/n\right)}}. \end{array}$$

Parameter *α* is related to the inverse of air entry value, the *n* parameter is related to the pore size distribution of the soil, and the *m* parameter is related to the asymmetry of the model. By substituting (1) into (7), we can write the volumetric water content form of the Van Genuchten model as

(8) $$\begin{array}{c} \theta =\theta_{r}+\frac{\theta_{s}-\theta_{r}}{{\left[1+{\left(\alpha \psi \right)}^{n}\right]}^{\left(1-1/n\right)}}. \end{array}$$

The Van Genuchten model has a complex form and relies on more fitting parameters than the models discussed above. However, it produces a continuous output in the unsaturated zone and provides a good description of the soil-water characteristic curve under most circumstances [23].

### 2.4. Lognormal Distribution Model

The last SWCC model considered is based on the model suggested by Kosugi [17]. This model was developed by applying a lognormal distribution law and its parameters are directly related to the soil pore radius distribution. The lognormal distribution model by Kosugi is described as follows:

(9) $$\begin{array}{c} \Theta =Q\left[\frac{\ln\psi /h_{m}}{\sigma }\right], \end{array}$$

where *Q* is related to the complementary error function, erfc, and defined as

(10) $$\begin{array}{c} Q\left(x\right)=\mathrm{erfc}\frac{\left(x/\sqrt{2}\right)}{2}. \end{array}$$

The model uses two fitting parameters, namely, *h*_*m*_ and *σ*. Parameter *h*_*m*_ is a capillary pressure head related to the median pore radius and *σ* is a dimensionless parameter related to the width of the pore radius distribution. By substituting (1) into (9), we can write the volumetric water content form of the lognormal distribution by Kosugi [17]:

(11) $$\begin{array}{c} \theta =\theta_{r}+\left(\theta_{s}-\theta_{r}\right)Q\left[\frac{\ln\psi /h_{m}}{\sigma }\right]. \end{array}$$

The lognormal distribution model has a more complex form because of the complementary error function present and thus it is difficult to use. The model does, however, have greater flexibility in terms of representing the soil-water characteristic curve in the wet and dry regions for all soil types [6].

### 2.5. Modification of Gardner's Model

In general, a good mathematical expression should have only a few parameters with clear physical meaning and easiness to use. Selection of the soil-water characteristic curve model and parameter determination was done by identifying a model that could accurately describe the soil-water characteristic curve under a broad range of conditions while being as simple as possible in order to facilitate its application on a region-wide scale. The Gardner model was judged to satisfy these criteria best and so was chosen as a starting-point.

Two basic parameters incorporated in Gardner's model are the air entry value (AEV) and pore size distribution, which are denoted as *a* and *n*, respectively. Here, the *a* parameter related to the air entry value of the soil is assumed to be

(12) $$\begin{array}{c} a=e^{-b}, \end{array}$$

where *b* is a fitting parameter which is related to the air entry value. Substituting (12) into (2) yields

(13) $$\begin{array}{c} \Theta =\frac{1}{1+e^{-b}\psi^{n}}. \end{array}$$

To facilitate the application of the model, it was rewritten by taking the exponential function of the right sides of the model. Rearranging (13) as a function of the exponential *e* gives

(14) $$\begin{array}{c} \Theta =\frac{1}{1+e^{\left(n\cdot \ln(\psi )-b\right)}}. \end{array}$$

**Figure 1:**
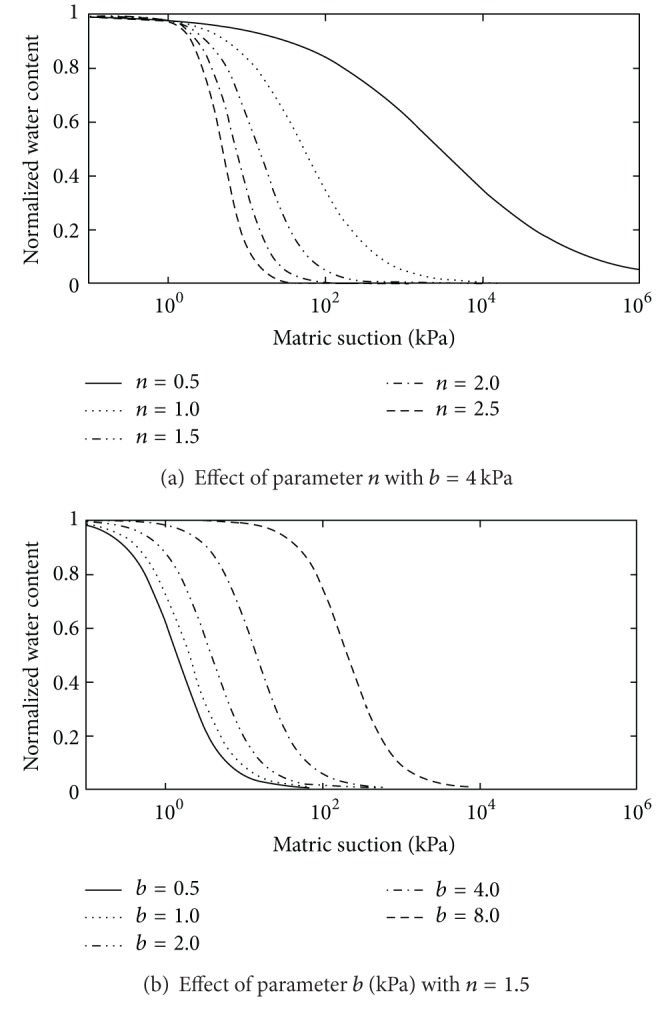
Effect of parameters *n* and *b* on the shape of the soil-water characteristic curve of the modified Gardner model.

The modified Gardner equation has two fitting parameters, namely, *b* and *n*. A parametric study was used to describe the fitting properties of the modified Gardner model.  Figure 1 shows a plot of the modified Gardner model when one parameter is changed and the others remain fixed. Only that feature of the curve related to the parameter being varied is affected. As a result, parameter *n* is related to the pore size distribution index of the soil (Figure 1(a)). The larger the value of *n*, the more uniform the pore size in the soil, and the steeper the slope of the curve. Parameter *b* locates the curve toward the higher or lower suction regions and has a unit of suction (Figure 1(b)). The *b* parameter does not affect the shape of the curve but shifts the curve towards the higher soil suction region as *b* increases.

The relationship between the volumetric water and suction can be obtained by substituting (1) into (14), yielding an expression for the soil-water characteristic curve:

(15) $$\begin{array}{c} \theta =\theta_{r}+\frac{\theta_{s}-\theta_{r}}{1+e^{\left(n\cdot \ln(\psi )-b\right)}}. \end{array}$$

The capability of the modified Gardner (MG) model was demonstrated for 12 soil types ranging from sand to clay. Prediction results were compared with the most widely used models by Brooks and Corey (BC), Van Genuchten (VG), and Kosugi (LN) to determine the MG model performance. These models are expressed as (5), (8), and (11), respectively. Other than *θ*_*s*_ and *θ*_*r*_, all models contain two fitting parameters. Therefore, for ease of scheduling, the fitting parameters are marked as P1 and P2.  Table 1 summarizes the parameters for the SWCC models evaluated.

## 3. Materials and Methods

### 3.1. Sources of Soil-Water Characteristic Curve Data

Twenty-four soil samples with soil-water characteristic data selected from the unsaturated soil hydraulic database (UNSODA 2.0) [24] are used to demonstrate the performance of the modified Gardner model. The selected data sets represent 12 soil textural classes in which each soil class is represented by two samples of data to be tested. The soils comprise sand, loamy sand, sandy loam, silty loam, silt, loam, sandy clay loam, silty clay loam, clay loam, sandy clay, silty clay, and clay. All these soils are identified in Table 2. These enable the modified Gardner model to validate and identify its parameters and compare them with the three most widely used models: BC, VG, and LN. The SWCCs in this study are presented in terms of volumetric water content, *θ* plotted on an arithmetical scale, and soil suction *ψ* plotted on a logarithmic scale.

### 3.2. Model Analysis

Optimization techniques are used to obtain the best-fit parameters for soil-water characteristic curve data sets. The curve fitting routine determines model parameters such that the mathematical function passes as close as possible to the experimental data points without necessarily going through any of the points. The fitting procedures for all 24 soil data sets were performed by nonlinear least-square analysis based on a trust-region algorithm method which employed the curve fitting tool in the MATLAB program. It is an iterative method starting with some initial values of the parameters. In the curve fitting tool program, the initial value of the parameters for the modified Gardner model is set to start with zero or 0.1 for all soil types. For the others (BC, VG, and LN model), the initial values for the model parameters in the iterative procedure were obtained by using reported literature values for the different soils.

There are various statistical measures which can be used to compare the fitting accuracy of the SWCC models. In this study, the root-mean-square error (RMSE) and the coefficient of determination (*R* ^2^) are used to help determine the best fit. The RMSE (m^3^m^−3^) statistic is an indicator of the overall error of the evaluated model function, with a value closer to zero indicating a better fit. The *R* ^2^ statistic is generally the best indicator of the fit quality. It is a measure of the linearity between observed and fitted data. The *R* ^2^ with a value approaching unity indicating that the observed and fitted data sets are linearly located around the line of perfect agreement or the fitted curve is of comparable shape as the observed curve.

The RMSE was expressed as

(16) $$\begin{array}{c} \text{RMSE}=\sqrt{\frac{1}{N}\left(\text{SSE}\right)}, \end{array}$$

where the SSE statistic is the least-squares error of the fit and defined as

(17) $$\begin{array}{c} \text{SSE}=\overset{N}{\underset{i=1}{\sum }}{\left[\theta_{i}^{\text{obs}}-\theta_{i}^{\text{fit}}\left(j\right)\right]}^{2}, \end{array}$$

where *j* is a parameter vector containing the unknowns that need to be estimated, *i* = 1, 2, …, *N*; *N* is the number of soil-water characteristic data for each soil sample, *θ*_*i*_ is the soil water content corresponding to the *i* data pair for each soil, and obs and fit denote observed and fitted values, respectively.

The value of *R* ^2^ reflects the proportion of the total sum of squares (SST) that is partitioned into the model sum of squares (SSM) since SST is equal to SSM plus SSE. Consider the following:

(18) $$\begin{array}{c} R^{2}=\frac{\text{SSM}}{\text{SST}}=1-\frac{\text{SSE}}{\text{SST}}. \end{array}$$

## 4. Results and Discussion

Table 3 shows the models' fitted parameters for various soil textural classes. In general, the values of *θ*_*r*_ for BC are lower than for the LN, VG, and MG models. The values of *θ*_*s*_ are very close to each other for the LN and MG models and for the VG and BC models, respectively. The parameter values shown in Table 3 can serve as useful initial values for researchers attempting to use one of the models.

The statistical measure values are shown in Table 4. To assist and facilitate the evaluation in determining the best model, a weightage is given to each fit. In this case, a weightage of one is given to the model that gave the smallest RMSE value (best fit) and a weightage of four is given to the model that gave the largest RMSE value (worst fit). These weightages were chosen as there are four equations ((5), (8), (11), and (15)) for comparison. The weightage is given in parentheses in Table 4 and a summary of the weightage evaluation results is shown in Table 5. From the total weightage in Table 5, it can be observed that the MG model performs much better than the three other models, as it has a weightage of 26 out of 96 total weightages representing all soil samples. The statistical analysis in terms of RMSE shows that the MG model led to the best fit of all the soil samples except for the loamy sand in soil sample 1 and the silt in soil sample 2 in which the VG model was better (Table 4). The RMSE values associated with the MG model for all soil samples ranged from 0.0005 to 0.0263, whereas for the LN model they varied between 0.0010 and 0.0273. The results clearly show that the RMSE values associated with the MG model are well below those of the LN model which earned 51 of the total weightages. It was closely followed by the VG model with 67 weightages, where the RMSE values associated with the VG model for all soil samples ranged from 0.0015 to 0.0257 and the BC model gained 96 of the total weightages, in which the RMSE varied between 0.0039 and 0.0294. Note that the fitting errors for the MG model are smaller than those for the other models. This is because of the better representation of the MG model for all soil samples. Results suggest that the MG model is most suitable for describing the observed data.

As regards the values of *R* ^2^, a similar trend could be observed. The MG model was the best model in terms of linearity, closely followed by the LN model, the VG model, and the BC model. In Table 4, the results obtained show that the *R* ^2^ values associated with the MG model are higher than those of the three other models for all soil samples except for the loamy sand in soil sample 1 and the silt in soil sample 2 for which the VG model result was better than that of the MG model. The *R* ^2^ values associated with the MG model for all soil samples ranged from 0.9585 to 0.9999, whereas for the LN model they varied between 0.9564 and 0.9997. The VG model's *R* ^2^ values for all soil samples ranged from 0.9382 to 0.9994, and the BC model's *R* ^2^ values varied between 0.8920 and 0.9961.

**Figure 2:**
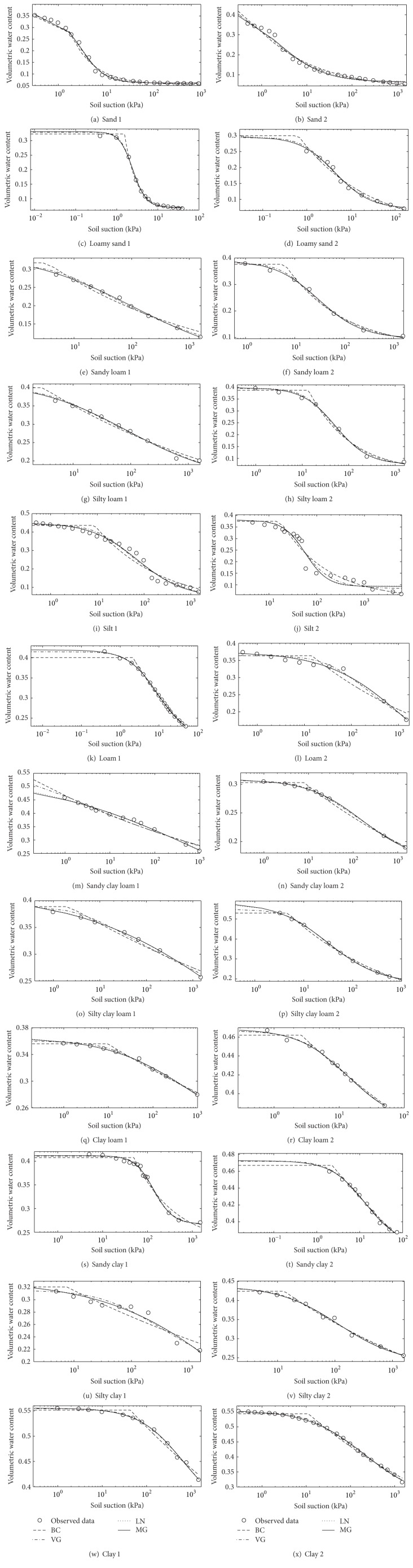
Observed and fitted soil-water characteristic curves (SWCCs) for sand ((a) and (b)), loamy sand ((c) and (d)), sandy loam ((e) and (f)), silt loam ((g) and (h)), silt ((i) and (j)), loam ((k) and (l)), sandy clay loam ((m) and (n)), silty clay loam ((o) and (p)), clay loam ((q) and (r)), sandy clay ((s) and (t)), silty clay ((u) and (v)), and clay ((w) and (x)). Numbers 1 and 2 represent soil samples 1 and 2, respectively, for each soil type.

To illustrate further the behavior of the four models compared in this study when fitted to the SWCC data for various soil types, the observed data and fitted curves are compared in Figures 2(a) to 2(x). With regard to the sand samples in Figures 2(a) and 2(b), the correspondence between observed and fitted SWCCs exhibited deviation for all models where they did not accurately match several points near saturation. All models still gave relatively good and realistic fits; however, the discontinuous character of the BC model did not seem to be problematic for the sand soils. With respect to the loamy sand in Figures 2(c) and 2(d), the MG, LN, and VG models showed very good fits to the observed data and no significant differences were observed between the three models in terms of their curves, although the VG model performed slightly better than the MG and LN models in the loamy sand of sample 1 (Figure 2(c)). The BC model missed the shape of the data near saturation because of its discontinuous character. Almost the same results were observed in sandy loam (Figures 2(e) and 2(f)) and silty loam (Figures 2(g) and 2(h)) soils. MG, LN, and VG models are very well matched to the observed data, though the MG performs better than the LN and VG models for both soils. The BC model typically had problems matching data in the transition point and did not accurately match some points near saturation, which decreased the flexibility of the curve. Although the BC model did not achieve a rate of success as high as the other models, it was able to match the observed data adequately. A similar trend could also be observed in loam soil (Figure 2(k)), sandy clay loam (Figure 2(n)), silty clay loam (Figures 2(o) and 2(p)), clay loam (Figures 2(q) and 2(r)), sandy clay (Figures 2(s) and 2(t)), silty clay (Figure 2(v)), and clay soils (Figures 2(w) and 2(x)). Slightly different results were obtained for the silt soil (Figure 2(i)), loam (Figure 2(l)), sandy clay loam (Figure 2(m)), and silty clay soil (Figure 2(u)); all models exhibit deviations in matching data in the transition point. All models still performed very well for all the samples in which they produced comparatively realistic fits. The same trend could also be observed in the silt soil in sample 2 (Figure 2(j)), where the correspondence between observed and fitted SWCCs exhibited deviation for all models. As can be seen in Figure 2(j), the only model that showed a better match to the data in the dry region was the BC model. Although all models did not accurately match several points of the available observed data in their data sets, they were still able to match the observed data in the extrapolation region adequately with a reasonable fit.

Overall, Figures 2(a) to 2(x) indicate that all models performed very well for all data sets except for the BC model because of its discontinuous character. If we compare the fitted curve of the MG and BC models, we can see that the MG model showed a better fit over the entire range of available observed data. The BC model typically had problems matching data in the transition point near saturation and mostly missed the shape of the data near saturation in almost all the soil samples, which decreased the flexibility of the curve. Therefore, this suggests that the MG model is more consistent when applied to different soils than is the BC model. As can be seen in Figures 2(a) to 2(x), the difference in the quality of fitting data between the MG and VG models is only marginal. As a result, out of 24 soil samples, the MG model fitted the observed data marginally better than the VG model in 22 soil samples. The VG model was slightly better than the MG model at fitting the other two soil samples (Figure 2(c) loamy sand 1 and Figure 2(j) silt 2). When we look at the fitted curve of the MG and LN models, both models predict almost identical curves and show much closeness to each other. However, if we compare the MG and LN models in terms of accuracy, we can see that the MG model showed a better fit for all 24 soil samples. These results indicate that the MG model can perform better than the LN model in terms of the goodness of fit for all soil types. Based on the data sets of soil in this study, it is therefore suggested that the exponential-based MG model performs much better than the BC, VG, and LN models and it is suitable for describing the SWCC for the 12 soil types.

## 5. Conclusion

In this paper, a simple modification of the Gardner model to form an exponential-based model has been performed to describe the soil-water characteristic curve. The exponential form of the modified Gardner model was then tested to show its performance and compared with the three most widely used models (BC, VG, and LN). Using a limited group of 24 soil samples from the UNSODA database which represents 12 different soil types, we analyzed and compared the four SWCC models with four fitting parameters. The models were evaluated in terms of their goodness of fit by means of statistical indexes. Results obtained show that out of 24 soil samples, the MG model fitted the observed data better than the LN, VG, and BC models for 22 samples. Therefore, the modified Gardner model was found to be the most consistent model fitting the observed data for the 12 soil types. The major achievement of the MG model is its ability to predict the entire range of available SWCC data with a wider range of soils and texture data. These results indicate that the exponential-based MG model performed better than the other models in terms of goodness of fit and matching the observed data. Nevertheless, additional research on testing the model performance under a complete range of water contents, from saturation to oven dryness, will be needed to evaluate the predictive capability of the model for the entire regions of the SWCC.

In summary, the advantages of the modified Gardner model are the following: the model parameters have a physical meaning which is related to the shape of the SWCC; the effect of one parameter can be distinguished from the effect of the other parameter; the model provides a wide range of flexibility to better fit data from a variety of soil types; and the model contains only four parameters. Given the results of the study, it is therefore suggested that the exponential-based MG model should be used as an alternative to the soil-water characteristic curve.

## Figures and Tables

**Table 1:** Parameters of the SWCC models.

Model	P1*	P2**
Brooks and Corey (BC)	*h*_*b*_	*λ*
Van Genuchten (VG)	*α*	*n*
Kosugi (LN)	*h*_*m*_	*σ*
Modified Gardner (MG)	*b*	*n*

*P1 has a unit of suction (kPa).

**P2 is a dimensionless parameter.

**Table 2:** Textural classes and UNSODA codes for soils used in this study.

Number	Textural class	UNSODA codes	
		Sample 1	Sample 2
1	Sand	4651	4660
2	Loamy sand	1062	1111
3	Sandy loam	1381	4171
4	Silty loam	1282	4180
5	Silt	4670	1330
6	Loam	2401	2614
7	Sandy clay loam	1184	2542
8	Silty clay loam	2593	3241
9	Clay loam	1123	1213
10	Sandy clay	1135	1174
11	Silty clay	1383	1361
12	Clay	2362	4680

**Table 3:** Model parameter values for various soil textural classes.

Soil type	Model	Soil sample 1				Soil sample 2			
		*θ*_*s*_	*θ*_*r*_	P1	P2	*θ*_*s*_	*θ*_*r*_	P1	P2
Sand	BC	0.3478	0.0540	1.2740	0.7945	0.4197	0.0246	0.3891	0.3328
	VG	0.3569	0.0591	0.4778	2.1840	0.4362	0.0486	1.5030	1.4840
	LN	0.3606	0.0624	3.2470	1.0170	0.4574	0.0678	2.1460	1.9630
	MG	0.3627	0.0611	1.9120	1.6260	0.4564	0.0641	0.6757	0.8432

Loamy sand	BC	0.3227	0.0650	1.5480	1.5300	0.2996	3.22*E* − 07	0.6394	0.2697
	VG	0.3289	0.0685	0.4861	3.1490	0.2941	0.0530	0.6094	1.5660
	LN	0.3294	0.0713	2.4610	0.6216	0.2976	0.0735	4.2480	1.5190
	MG	0.3308	0.0700	2.3920	2.6570	0.2972	0.0704	1.5940	1.0780

Sandy loam	BC	0.3170	4.85*E* − 07	3.3910	0.1472	0.3760	0.0569	6.2370	0.3708
	VG	0.3144	2.14*E* − 05	0.1303	1.1830	0.3835	0.0788	0.0981	1.5340
	LN	0.3377	0.0521	113.30	3.3450	0.3883	0.1001	29.890	1.6520
	MG	0.3475	0.0430	2.1290	0.4512	0.3915	0.0962	3.3350	0.9795

Silty loam	BC	0.3995	1.43*E* − 05	3.0280	0.1083	0.3874	0.0417	13.670	0.4824
	VG	0.3995	4.13*E* − 05	0.1736	1.1280	0.3953	0.0601	0.0439	1.7030
	LN	0.4114	0.1537	86.940	2.8870	0.3968	0.0787	51.180	1.3300
	MG	0.4194	0.1446	2.3500	0.5241	0.3985	0.0747	4.8500	1.2260

Silt	BC	0.4358	1.73*E* − 07	8.761	0.3027	0.3750	0.0374	17.350	0.4392
	VG	0.4414	3.16*E* − 07	0.0542	1.4060	0.3803	0.0831	0.0266	2.0980
	LN	0.4476	0.0529	70.590	1.8720	0.3790	0.0943	57.900	0.8778
	MG	0.4510	0.0464	3.7360	0.8711	0.3808	0.0927	7.6270	1.8820

Loam	BC	0.4007	2.81*E* − 04	2.1230	0.1795	0.3637	1.05*E* − 09	14.440	0.1330
	VG	0.4151	0.1499	0.2749	1.4650	0.3642	9.13*E* − 06	0.0193	1.2060
	LN	0.4153	0.2049	9.2920	1.4010	0.3714	7.30*E* − 09	1308.0	3.0400
	MG	0.4197	0.1994	2.4790	1.1070	0.3752	1.35*E* − 06	3.9000	0.5485

Sandy clay loam	BC	0.5337	1.13*E* − 10	0.1632	0.0742	0.3030	7.63*E* − 04	7.917	0.0882
	VG	0.5322	2.35*E* − 08	5.0510	1.0760	0.3069	4.56*E* − 05	0.0739	1.1030
	LN	0.5332	1.26*E* − 05	882.40	6.5790	0.3078	0.1642	167.20	2.3470
	MG	0.5337	2.47*E* − 07	1.6910	0.2511	0.3089	0.1657	3.5820	0.7081

Silty clay loam	BC	0.3890	7.50*E* − 08	2.2220	0.0569	0.5300	1.98*E* − 05	4.6970	0.1883
	VG	0.3902	1.06*E* − 06	0.2363	1.0670	0.5488	0.1355	0.1029	1.4110
	LN	0.4070	8.30*E* − 05	11740	6.0330	0.5723	0.1881	31.560	1.8770
	MG	0.4151	0.0019	2.4390	0.2673	0.5838	0.1771	2.8110	0.8148

Clay loam	BC	0.3561	0.0373	9.6590	0.0564	0.4620	0.1249	2.6300	0.0838
	VG	0.3600	9.97*E* − 05	0.0799	1.0570	0.4665	0.3425	0.1818	1.4560
	LN	0.3653	0.1338	3982.0	4.3040	0.4666	0.3696	13.930	1.3990
	MG	0.3653	0.2126	3.0920	0.4859	0.4687	0.3680	2.9330	1.1190

Sandy clay	BC	0.4074	0.2244	49.820	0.4754	0.4669	0.0547	2.2820	0.0647
	VG	0.4103	0.2647	0.0100	2.4730	0.4718	0.3427	0.2168	1.4010
	LN	0.4106	0.2684	139.50	0.8112	0.4723	0.3734	13.090	1.4870
	MG	0.4116	0.2672	9.9810	2.0240	0.4725	0.3724	2.8360	1.0950

Silty clay	BC	0.3206	9.12*E* − 08	7.5270	0.0628	0.4243	0.0065	13.100	0.1091
	VG	0.3150	9.28*E* − 04	0.0208	1.1060	0.4331	0.1635	0.0442	1.2550
	LN	0.3266	1.27*E* − 05	9142.0	4.2280	0.4350	0.2390	117.50	1.9140
	MG	0.3315	1.24*E* − 05	3.5180	0.3942	0.4382	0.2357	3.9630	0.8314

Clay	BC	0.5513	1.36*E* − 04	43.010	0.0743	0.5437	2.99*E* − 07	11.250	0.1023
	VG	0.5543	3.08*E* − 05	0.0084	1.1130	0.5502	5.73*E* − 04	0.0549	1.1210
	LN	0.5545	0.3147	949.70	2.0800	0.5531	0.2468	251.30	2.5180
	MG	0.5555	0.3466	5.6740	0.8757	0.5559	0.2600	3.6070	0.6748

**Table 4:** Statistical measures of the models for various soil textural classes.

Soil type	Model	Soil sample 1			Soil sample 2		
		*R* ^2^	RMSE	Wt.*	*R* ^2^	RMSE	Wt.*
Sand	BC	0.9878	0.0147	(4)	0.9765	0.0216	(4)
	VG	0.9958	0.0086	(3)	0.9890	0.0147	(3)
	LN	0.9964	0.0080	(2)	0.9909	0.0134	(2)
	MG	0.9972	0.0071	(1)	0.9923	0.0124	(1)

Loamy sand	BC	0.9961	0.0074	(4)	0.9784	0.0125	(4)
	VG	0.9973	0.0061	(1)	0.9898	0.0092	(3)
	LN	0.9962	0.0073	(3)	0.9911	0.0086	(2)
	MG	0.9971	0.0064	(2)	0.9923	0.0080	(1)

Sandy loam	BC	0.9764	0.0113	(4)	0.9838	0.0192	(4)
	VG	0.9931	0.0066	(3)	0.9935	0.0122	(3)
	LN	0.9980	0.0035	(2)	0.9966	0.0088	(2)
	MG	0.9982	0.0034	(1)	0.9974	0.0078	(1)

Silty loam	BC	0.9779	0.0120	(4)	0.9813	0.0220	(4)
	VG	0.9901	0.0080	(3)	0.9939	0.0126	(3)
	LN	0.9938	0.0064	(2)	0.9965	0.0096	(2)
	MG	0.9940	0.0062	(1)	0.9973	0.0083	(1)

Silt	BC	0.9591	0.0294	(4)	0.9529	0.0288	(4)
	VG	0.9797	0.0207	(3)	0.9624	0.0257	(1)
	LN	0.9840	0.0188	(2)	0.9575	0.0273	(3)
	MG	0.9857	0.0178	(1)	0.9608	0.0263	(2)

Loam	BC	0.9909	0.0063	(4)	0.9287	0.0191	(4)
	VG	0.9990	0.0021	(3)	0.9705	0.0131	(3)
	LN	0.9993	0.0018	(2)	0.9826	0.0094	(2)
	MG	0.9995	0.0015	(1)	0.9864	0.0083	(1)

Sandy clay loam	BC	0.9793	0.0120	(4)	0.9906	0.0049	(4)
	VG	0.9810	0.0109	(3)	0.9991	0.0015	(3)
	LN	0.9954	0.0057	(2)	0.9996	0.0010	(2)
	MG	0.9956	0.0053	(1)	0.9999	0.0005	(1)

Silty clay loam	BC	0.9613	0.0105	(4)	0.9959	0.0104	(4)
	VG	0.9795	0.0077	(3)	0.9994	0.0038	(3)
	LN	0.9980	0.0027	(2)	0.9997	0.0028	(2)
	MG	0.9986	0.0022	(1)	0.9998	0.0024	(1)

Clay loam	BC	0.9631	0.0064	(4)	0.9837	0.0039	(4)
	VG	0.9869	0.0038	(3)	0.9932	0.0025	(3)
	LN	0.9942	0.0025	(2)	0.9934	0.0025	(2)
	MG	0.9953	0.0023	(1)	0.9942	0.0024	(1)

Sandy clay	BC	0.9712	0.0096	(4)	0.9794	0.0049	(4)
	VG	0.9928	0.0048	(3)	0.9939	0.0027	(3)
	LN	0.9931	0.0047	(2)	0.9953	0.0023	(2)
	MG	0.9942	0.0043	(1)	0.9965	0.0020	(1)

Silty clay	BC	0.8920	0.0129	(4)	0.9850	0.0094	(4)
	VG	0.9382	0.0105	(3)	0.9924	0.0067	(3)
	LN	0.9564	0.0088	(2)	0.9932	0.0063	(2)
	MG	0.9585	0.0086	(1)	0.9936	0.0061	(1)

Clay	BC	0.9806	0.0077	(4)	0.9859	0.0096	(4)
	VG	0.9968	0.0031	(2)	0.9982	0.0035	(3)
	LN	0.9966	0.0032	(3)	0.9989	0.0028	(2)
	MG	0.9971	0.0030	(1)	0.9989	0.0027	(1)

*Values in parentheses are the weightage (Wt.) where a value of one indicates least RMSE value and a value of four indicates largest RMSE value for the data set.

**Table 5:** Summary of weightage evaluation results for all data sets.

Model	Weightage scores for all data sets								Total weightage
	Soil sample 1				Soil sample 2				
	(1)	(2)	(3)	(4)	(1)	(2)	(3)	(4)	
BC	—	—	—	12	—	—	—	12	96
VG	1	1	10	—	1	—	11	—	67
LN	—	10	2	—	—	11	1	—	51
MG	11	1	—	—	11	1	—	—	26
